# Multilevel Determinants of COVID-19 Vaccine Uptake Among South Asian Ethnic Minorities in Hong Kong: Cross-sectional Web-Based Survey

**DOI:** 10.2196/31707

**Published:** 2021-11-09

**Authors:** Akansha Singh, Angel Hor Yan Lai, Jingxuan Wang, Saba Asim, Paul Shing-Fong Chan, Zixin Wang, Eng Kiong Yeoh

**Affiliations:** 1 JC School of Public Health and Primary Care Faculty of Medicine The Chinese University of Hong Kong Hong Kong China (Hong Kong); 2 Centre for Health Systems and Policy Research JC School of Public Health and Primary Care, Faculty of Medicine The Chinese University of Hong Kong Hong Kong China (Hong Kong); 3 Department of Applied Social Science The Hong Kong Polytechnic University Hong Kong China (Hong Kong)

**Keywords:** COVID-19, South Asian ethnic minorities, COVID-19 vaccination, uptake, cultural and religious reasons for vaccine hesitancy, perceptions, information exposure on social media, influence of peers, socioecological model, Hong Kong

## Abstract

**Background:**

The COVID-19 pandemic continues to have a disproportionate effect on ethnic minorities. Across countries, greater vaccine hesitancy has been observed among ethnic minorities. After excluding foreign domestic helpers, South Asians make up the largest proportion of ethnic minorities in Hong Kong. It is necessary to plan for COVID-19 vaccination promotional strategies that cater to the unique needs of South Asians in Hong Kong.

**Objective:**

This study investigated the prevalence of COVID-19 vaccine uptake among a sample of South Asians in Hong Kong. We examined the effects of sociodemographic data and factors at individual level (perceptions), interpersonal level (information exposure on social media), and sociostructural level (cultural) based on the socioecological model.

**Methods:**

A cross-sectional web-based survey was conducted on May 1-31, 2021. Participants were South Asian people aged 18 years or older living in Hong Kong; able to comprehend English, Hindi, Nepali, or Urdu; and having access to a smartphone. Three community-based organizations providing services to South Asians in Hong Kong facilitated the data collection. The staff of the community-based organizations posted the study information in WhatsApp groups involving South Asian clients and invited them to participate in a web-based survey. Logistic regression models were fit for data analysis.

**Results:**

Among 245 participants, 81 (33.1%) had taken at least one dose of the COVID-19 vaccine (one dose, 62/245, 25.2%; and both doses, 19/245, 7.9%). After adjusting for significant background characteristics, cultural and religious reasons for COVID-19 vaccine hesitancy were associated with lower COVID-19 vaccine uptake (adjusted odds ratio [AOR] 0.83, 95% CI 0.71-0.97; *P*=.02). At the individual level, having more positive attitudes toward COVID-19 vaccination (AOR 1.31, 95% CI 1.10-1.55; *P*=.002), perceived support from significant others (AOR 1.29, 95% CI 1.03-1.60; *P*=.03), and perceived higher behavioral control to receive COVID-19 vaccination (AOR 2.63, 95% CI 1.65-4.19; *P*<.001) were associated with higher COVID-19 vaccine uptake, while a negative association was found between negative attitudes and the dependent variable (AOR 0.73, 95% CI 0.62-0.85; *P*<.001). Knowing more peers who had taken the COVID-19 vaccine was also associated with higher uptake (AOR 1.39, 95% CI 1.11-1.74; *P*=.01). At the interpersonal level, higher exposure to information about deaths and other serious conditions caused by COVID-19 vaccination was associated with lower uptake (AOR 0.54, 95% CI 0.33-0.86; *P*=.01).

**Conclusions:**

In this study, one-third (81/245) of our participants received at least one dose of the COVID-19 vaccine. Cultural or religious reasons, perceptions, information exposure on social media, and influence of peers were found to be the determinants of COVID-19 vaccine uptake among South Asians. Future programs should engage community groups, champions, and faith leaders, and develop culturally competent interventions.

## Introduction

The COVID-19 pandemic is an ongoing threat [1]. COVID-19 vaccination and other behavioral preventive measures can help to eradicate this pandemic. The Hong Kong government procured 2 types of COVID-19 vaccines (Sinovac-Biotech and BioNTech-Fosun Pharma) and implemented a free-of-charge territory-wide vaccination program to all Hong Kong residents aged 16 years or older. The vaccination services were provided through community vaccination centers, designated public and private clinics, and outreach vaccination services at residential care homes or nursing homes [2]. The program aimed to cover the entire Hong Kong population. During the study period (May 1-31, 2021), priorities to receive COVID-19 vaccination were given to the following groups of Hong Kong residents: (1) individuals aged 30 years or older and caregivers of older adults aged more than 70 years; (2) personnel in health care settings and those participating in antiepidemic-related work; (3) residents and staff of residential care homes for the older adults/persons with disabilities; (4) personnel maintaining critical public services; (5) those providing cross-boundary transportation or working at control points/ports; (6) staff of food and beverage premises; (7) staff of local public transportation operators; (8) registered construction workers; (9) staff of property management; (10) teachers and school staff; (11) staff of tourism industry; (12) staff of scheduled premises under the Prevention and Control of Disease Regulation (eg, staff of fitness centers, beauty parlors); (13) students studying outside Hong Kong (aged 16 years or older); and (14) domestic helpers [2]. The latest estimate shows that at least 70% immune individuals would be necessary to achieve herd immunity for COVID-19 [3]. The number of people who received at least one dose of COVID-19 vaccine increased from 936,400 on May 1, 2021 to 1,379,400 on May 31, 2021, accounting for 12.3% and 18.2% of the entire population in Hong Kong, respectively [4]. However, it will take about 1 year for Hong Kong to achieve herd immunity based on the current progress.

Across countries, COVID-19 pandemic continues to have a disproportionate effect on ethnic minorities, with higher COVID-19 morbidity and mortality and greater adverse socioeconomic consequences [5]. With mass COVID-19 vaccination programs in progress, disparities in its uptake may aggravate the vulnerability of ethnic minorities. Across countries, greater vaccine hesitancy has been observed among people from some ethnic minorities [5-7]. In the United Kingdom, vaccine hesitancy was higher among Black, Bangladeshi, and Pakistani people compared with people from a White ethnic background [8]. Two other studies reported lower COVID-19 vaccine uptake rates among ethnic minorities who were older than 80 years (20.5% among Black people vs 42.5% among White people) and those who were health care workers (70.9% White people, 58.5% South Asians, and 36.8% Black people) [9,10]. It is hence important to understand and address the disparities in COVID-19 vaccination among ethnic minorities.

In Hong Kong, the ethnic minority population increased significantly by 70.8% from 2006 to 2016 and accounted for 8.0% (n=584,383) of the entire population (Census, 2016) [11]. After excluding foreign domestic helpers (most of them are Filipinos and Indonesians), South Asians, including Indians, Pakistanis, Nepalis, Bangladeshis, and Sri Lankans make up the largest proportion of the ethnic minorities in Hong Kong (n=85,875, accounting for 1.2% of the entire Hong Kong population) [11]. A recent study found that health system responsiveness reported by South Asians was lower than that reported by Chinese patients for both outpatient and inpatient services in Hong Kong [12]. The largest disparity in the responsiveness was shown in the communication barriers experienced by South Asians owing to cultural and language differences between South Asian patients and local health care service providers [12]. During hospitalization, South Asian inpatients perceived limited access to community support in comparison with the Chinese inpatients [12]. There is also a lack of autonomy in decision-making and choice of health care providers experienced by South Asians [12]. Health professionals in Hong Kong also indicated barriers in their delivery of services to the ethnic minorities, including inadequate dissemination of appropriate information, insufficient provision of cross-cultural care education and training, inadequate availability of public primary care services, and presence of bias and discrimination among hospital staff [12]. Cultural differences between ethnic minorities and health service providers affect patient-provider interactions and health care quality, resulting in mistrust of government and health authorities, which in turn becomes an obstacle for the COVID-19 vaccination program [5]. With an increasing population of South Asians in Hong Kong, it is necessary to plan for COVID-19 vaccination promotional strategies that cater to their unique needs.

This study applied the socioecological model as the conceptual framework, which considered determinants of a health behavior at the individual, interpersonal, and sociostructural levels [13]. An intervention addressing factors at multiple levels is more likely to be successful in changing behavior [13]. At the sociostructural level, previous studies have pointed out the association between cultural/religious belief and COVID-19 vaccine hesitancy. A Malaysian study showed that 20.8% and 6.8% of the ethnic minorities believed that their COVID-19 vaccine hesitancy was caused by concerns regarding religious and cultural factors, respectively [14]. News reports also suggested that there were concerns among Muslims over the halal status of the COVID-19 vaccine [15], and the beliefs that COVID-19 should be healed by God and the body is sacred were cited as reasons to refuse the COVID-19 vaccine [16]. Because a large percentage of the South Asians in Hong Kong are Muslims, there is a need to understand the effects of religious beliefs on COVID-19 vaccine uptake, so that policymakers can target this specific population for the relevant COVID-19 vaccine promotional strategy. Limited evidence is available to date to inform us on the cultural effect of COVID-19 vaccine uptake in Hong Kong.

At the interpersonal level, misinformation related to COVID-19 vaccination threatened vaccine uptake [17]. The government used to report deaths after COVID-19 vaccination, regardless of the existence of the direct causality evidence. Such information exposure might inhibit the motivation to receive COVID-19 vaccination among South Asians as they will associate COVID-19 vaccination directly with deaths. For example, public’s concerns about vaccine safety increased substantially after media reported deaths after COVID-19 vaccination, which resulted in a drop in the proportion of people turning up for their vaccination appointment in Hong Kong (from >90% to 72%) [2]. However, communication with peers may be effective among South Asians owing to higher level of rapport between people of the same ethnicity [18]. The Social Learning Theory posits that observation of peers has a major influence on people’s health behaviors. Therefore, vaccinated peers may play important roles as volunteers in future programs promoting COVID-19 vaccination among South Asians by sharing their positive experience. Peers’ experiences related to COVID-19 vaccination might have a strong influence on South Asians’ decision to receive such vaccinations. Based on these observations, socialization, in terms of receiving vaccine-related information or interpersonal interaction, is assumed to affect vaccine uptake.

At the individual level, the Theory of Planned Behavior (TPB) postulates that in order to perform a behavior, one would evaluate the pros and cons of the behavior (positive and negative attitudes), consider whether their significant others would support such behavior (perceived subjective norm), and appraise how much control one has over the behavior (perceived behavioral control) [19]. The TPB was commonly used to explain a health behavior and guide the behavioral intervention [20,21]; it has been used to explain willingness to receive COVID-19 vaccination among Chinese people [22]. Studies conducted among the Hong Kong general population identified the perceived pros and cons that were associated with the willingness to receive COVID-19 vaccination. Pros included perceived greater risk and severe consequences of infection, perceived higher effectiveness of COVID-19 vaccines, perceived greater impact of COVID-19 vaccination in pandemic control, perceived larger proportion of general public and acquaintance would take up such vaccination, being recommended by physicians and family members, and perceived higher behavioral control to take up the vaccination [23-26]. Concerns related to side effects and access issues as well as difficulties in choosing one out of the available COVID-19 vaccines were commonly mentioned as cons against vaccination [23-26]. These perceptions were considered by this study. To our knowledge, there is a dearth of studies investigating the determinants of COVID-19 vaccine uptake among ethnic minorities in the Asian region. To address the knowledge gaps, this study investigated the prevalence of COVID-19 vaccine uptake among a sample of South Asians in Hong Kong. We examined the effects of factors, including sociodemographic data, and all 3 levels of factors based on the socioecological model.

## Methods

### Study Design

We conducted a cross-sectional web-based survey of 245 South Asian adults in Hong Kong, China on May 1-31, 2021.

### Participants and Data Collection

**Figure 1:**
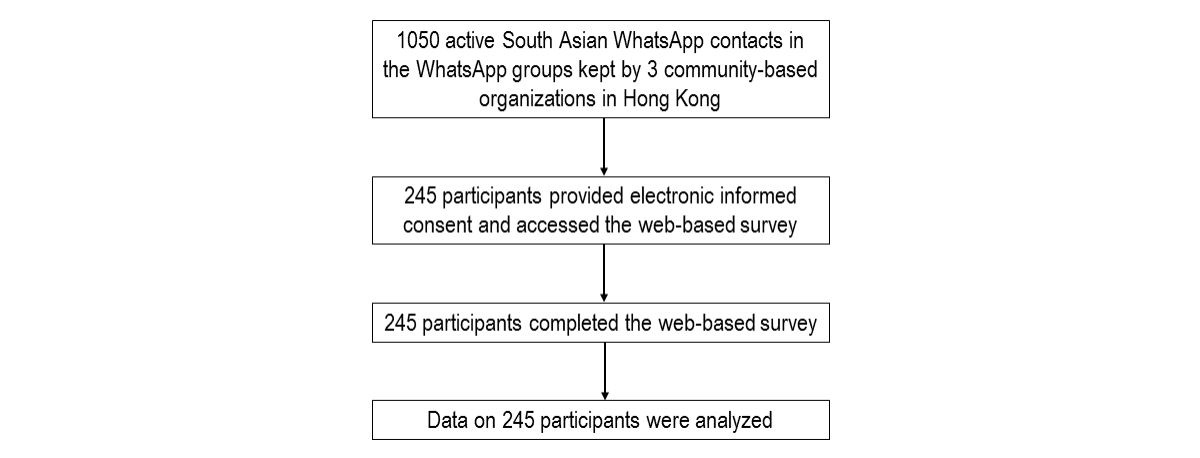
Flowchart of data collection.

Participants were South Asian people aged 18 years or older living in Hong Kong; able to comprehend English, Hindi, Nepali, or Urdu; and having access to a smartphone. Three community-based organizations (CBOs) in Hong Kong facilitated the data collection. These CBOs provide a wide range of services to ethnic minorities, including early childhood education, child rehabilitation, school social work, treatment and social rehabilitation for drug abusers, home care for older adults, employee assistance and development, health check-up, and health education. However, their services do not include provision of COVID-19 vaccination. The CBOs keep a list of South Asian people using their services and WhatsApp groups involving CBO staff, and these service users are established. There are around 1050 active South Asian people in the WhatsApp groups held by the CBOs. CBO staff posted the study information in the WhatsApp groups and invited South Asian people in these groups to participate in the web-based survey. A link to access a web-based self-administered survey was also posted in the WhatsApp groups. Through the link, participants first selected the language of the survey (English, Hindi, Nepali, or Urdu) and then accessed an electronic consent form. On the form, they read the study information and a statement indicating that the information collected by the survey was only used for scientific research purposes and would be kept strictly confidential. Participation was completely voluntary, and refusal would have no consequences. After clicking “I agree” on the electronic consent form, they could start the web-based survey. We developed the questionnaire by using Google forms, a commonly used web-based survey platform. Each WhatsApp account was only allowed to access the web-based questionnaire once to avoid duplicate responses. The survey had about 100 items, which took about 20 minutes to complete. The Google form performed a completeness check before each questionnaire was submitted. Participants were able to review and change their responses before submission. Participants were asked to leave an address at the end of the survey. The procedure of data collection is shown in Figure 1. A supermarket coupon of HK $50 (US $6.5) was sent to the participants by mail upon completion. All data were stored in the web-based server of the Google form and protected by a password. Only the corresponding author had access to the database.

### Ethics Approval and Consent to Participate

Informed consent was obtained from all the participants involved in this study. Ethical approval was obtained from the Survey and Behavioral Research Committee of the Chinese University of Hong Kong (Reference SBRE-20-534).

### Sample Size Planning

Our target sample size was 250. Given a statistical power of .80 and an α value of .05 and assuming the COVID-19 vaccine uptake in the reference group (without a facilitating condition) to be 10%-20%, the sample size could detect the smallest odds ratio of 2.23 between people with and without the facilitating conditions (PASS 11.0, NCSS LLC).

### Measurements

#### Development of the Questionnaire

A panel consisting of public health researchers, social workers, health psychologists, representatives from South Asian communities, and CBO workers was formed to develop the questionnaire. Bilingual researchers with master’s degrees translated the English version of the questionnaire into Hindi, Nepali, or Urdu. The agreed versions were back translated into English by independent bilingual researchers to ensure linguistic equivalence. The questionnaire was tested on the readability and length among 20 South Asians speaking English, Hindi, Nepali, or Urdu. All participants in the pilot testing agreed that the wordings of the questions were appropriate and easy to understand. However, 15 of them commented that the questionnaire was too long. The panel trimmed down the questionnaire from 130 items to 100 items. Thereafter, the participants were invited to comment on the length of the revised questionnaire, and they all agreed that the length was acceptable. The panel then finalized the questionnaire for the actual survey. The English version of the questionnaire is in Multimedia Appendix 1.

#### Background Characteristics

Participants were asked to report on sociodemographic data and living arrangements (eg, number of family members living with them, whether they were living with children younger than 18 years or older adults aged ≥60 years). Participants also reported compliance to personal preventive behaviors in the past month, including frequency of wearing facemasks when having close contact with others in workplaces and other public spaces and sanitizing hands after returning from public spaces or touching public installation (response categories: every time, often, sometimes, and never). Two physical distancing behaviors were also measured (whether they avoided social or meal gatherings with people who they do not live with and crowded places in the past month). Same measurements of personal preventive behaviors and physical distancing were used in published studies [19,22,27,28].

#### COVID-19 Vaccine Uptake

Participants reported whether they had taken any COVID-19 vaccine. Some supplementary information was collected from the vaccinated participants, including number of doses and types of COVID-19 vaccines received, presence of side effects, and severity of such side effects.

#### Individual-, Interpersonal-, and Sociostructural-Level Variables Related to COVID-19 Vaccination

At the individual level, positive attitudes toward COVID-19 vaccination were measured by the validated Chinese version of the Positive Attitude Scale [19]. The original scale has 5 items, and the Cronbach α was .84 [19]. The scale was adapted by replacing “China” in the original scale with “Hong Kong.” Perceived subjective norm related to COVID-19 vaccination was measured by the validated Chinese version of the Subjective Norm Scale [19]. The Cronbach α of the original 2-item scale was .85. We added 1 more item “your friends from South Asia would support you to receive COVID-19 vaccination” to the original scale. Regarding perceived behavioral control related to COVID-19 vaccination, we added 1 more item “you are confident to receive COVID-19 vaccination in the next six months if you want to” to the validated single-item measurement [19]. One scale (6 items) was constructed for this study to measure negative attitudes toward COVID-19 vaccination (eg, the side effects of COVID-19 vaccines in the long run is unclear). The response categories to the aforementioned scale items were 1 (disagree), 2 (neutral), and 3 (agree). In addition, one single item was constructed to measure the descriptive norm related to COVID-19 vaccination “Among South Asians you know who are living in Hong Kong, how many of them have already taken up COVID-19 vaccines?” (response categories: 1=none/not sure, 2=1-2, 3=3-5, 4=6-10, and 5=more than 10). At the interpersonal level, the frequency of exposing to negative information related to COVID-19 vaccination on social media (eg, Facebook, Twitter, Flicker, TikTok) in the past were measured (response categories: 0=almost never, 1= seldom, 2=sometimes, and 3=always). Participants were also asked about whether they heard about any South Asians who experienced serious side effects after taking up COVID-19 vaccines. At the sociostructural level, 5 items measured cultural and religious reasons for COVID-19 vaccine hesitancy (eg, you are concerned about the halal status of the COVID-19 vaccines and the body is sacred; it should not receive certain chemicals or blood or tissues from animals). The Cultural and Religious Barrier Scale was constructed by summing up individual item scores. In addition, 2 items measured how much confidence they had in Hong Kong’s health care system and how much they trusted the Hong Kong government regarding COVID-19 control (response categories: from 1=not at all to 10=extremely).

### Statistical Analysis

Self-reported uptake of any COVID-19 vaccine was the dependent variable. Univariate logistic regression models first assessed the significance of the association between background characteristics and the dependent variable. We fitted a single logistic regression model to obtain adjusted odds ratios (AOR), which involved one of the independent variable of interest and all background characteristics with *P* values less than .05 in univariate analysis. The same approach to obtain AOR was commonly used in published studies [19,20,22,27,28]. There was no missing value for the participants who completed the survey. Therefore, missing value analysis was not performed. SPSS version 26.0 (IBM Corp) was used for data analysis, with *P*<.05 considered statistically significant.

## Results

### Background Characteristics

Among the 245 participants who completed the web-based survey, 83 (33.9%) were Indians, 89 (36.3%) were Pakistanis, 52 (21.2%) were Nepalis, and 21 (8.6%) were from other ethnicity groups. The majority of the participants were younger than 40 years, females, married or cohabited with a partner, and with tertiary education. About 29.4% (72/245) of the participants were Hong Kong permanent residents and had a full-time job. Participants reported good compliance with personal preventive behaviors and physical distancing behaviors in the past month (Table 1).

**Table 1:** Background characteristics of the South Asians who completed the web-based survey in Hong Kong on May 1-31, 2021 (N=245).

Characteristics				Values, n (%)
**Sociodemographic characteristics**				
	**Age group (years)**			
		18-29	83 (33.9)	
		30-39	100 (40.8)	
		40-49	55 (22.4)	
		≥50	7 (2.9)	
	**Gender**			
		Male	83 (33.9)	
		Female	162 (66.1)	
	**Relationship status**			
		Currently single	90 (36.7)	
		Married or cohabiting with a partner	155 (63.3)	
	**Ethnicity**			
		Indian	83 (33.9)	
		Pakistani	89 (36.3)	
		Nepali	52 (21.2)	
		Other ethnicity groups	21 (8.6)	
	**Permanent residents of Hong Kong**			
		Yes	72 (29.4)	
		No	173 (70.6)	
	**Highest education level attained**			
		Junior high or below	35 (14.3)	
		Senior high or equivalent	64 (26.1)	
		College or university	103 (42.0)	
		Postgraduate	43 (17.6)	
	**Employment status**			
		Full-time	85 (34.7)	
		Part-time/self-employed/housewife/unemployed/retired/students	160 (65.3)	
	**Family members living with the participant**			
		0	15 (6.1)	
		1-2	44 (18.0)	
		3-4	124 (50.6)	
		≥5	62 (25.3)	
	**Living with an older adult aged ≥60 years**			
		No	69 (28.2)	
		Yes	176 (71.8)	
	**Living with a child aged <18 years**			
		No	181 (73.9)	
		Yes	64 (26.1)	
	**Having at least one chronic condition**			
		No	221 (90.2)	
		Yes	24 (9.8)	
**Compliance to personal preventive behaviors and physical distancing**				
	**Frequency of facemask wearing when in proximity with other people in workplace**			
		Never/sometimes/often	20 (8.2)	
		Every time	225 (91.8)	
	**Frequency of facemask wearing in public spaces/transportations other than workplaces**			
		Never/sometimes/often	46 (18.8)	
		Every time	199 (81.2)	
	**Sanitizing hands after returning from public spaces or touching public installation**			
		Never/sometimes/often	60 (24.5)	
		Every time	185 (75.5)	
	**Avoiding social/meal gathering with other people who do not live together**			
		No	65 (26.5)	
		Yes	180 (73.5)	
	**Avoiding crowded places**			
		No	59 (24.1)	
		Yes	186 (75.9)	

### COVID-19 Vaccine Uptake

Among the participants, 33.1% (81/245) had received at least one dose of COVID-19 vaccine (one dose, 62/245, 25.2%; both doses, 19/245, 7.9%). Among the vaccinated participants (81/245), most of them chose mRNA vaccines manufactured by the BioNTech-Fosun Pharma and received vaccination at community vaccination centers. The side effects of the COVID-19 vaccination were reported by 64 participants (79%), and most of the side effects were very mild/mild (Table 2).

**Table 2:** Perceptions and influences of social media and peers related to COVID-19 vaccination among South Asians who completed the web-based survey in Hong Kong on May 1-31, 2021 (N=245).

Characteristics							Values
**Uptake of at least one dose of COVID-19 vaccine, n (%)**							
	No					164 (66.9)	
	Yes					81 (33.1)	
**Individual-level factors, n (%)**							
	**Perceptions related to COVID-19 vaccination based on the Theory of Planned Behavior**						
		**Positive attitudes toward COVID-19 vaccination (agree)**					
			COVID-19 vaccination is highly effective in protecting you from COVID-19		130 (53.1)		
			Taking up COVID-19 vaccination is highly effective in protecting your family members against COVID-19		136 (55.5)		
			Taking up COVID-19 vaccination can facilitate resumption of cross-boundary travelling		164 (66.9)		
			Taking up COVID-19 vaccination can contribute to the control of COVID-19 in Hong Kong		159 (64.9)		
			Hong Kong will have adequate supply of COVID-19 vaccination		178 (72.7)		
			Positive Attitude Scale^a^, mean (SD)		12.7 (2.3)		
		**Negative attitudes toward COVID-19 vaccination (agree)**					
			COVID-19 vaccines will have severe side effects		69 (28.2)		
			The side effects of COVID-19 vaccines in the long run is unclear		104 (42.4)		
			The protection of COVID-19 vaccines will only last for a short time		54 (22.0)		
			It is difficult for you to register for COVID-19 vaccination		21 (8.6)		
			There is a lack of information related to the COVID-19 vaccination program in my mother tongue		79 (32.2)		
			You do not know which type of COVID-19 vaccine is the most suitable for you		105 (42.9)		
			Negative Attitude Scale^b^, mean (SD)		11.9 (2.3)		
		**Perceived subjective norm related to COVID-19 vaccination (agree)**					
			Doctors and nurses would support you to receive COVID-19 vaccination		121 (49.4)		
			Your family members will support you to receive COVID-19 vaccination		168 (68.6)		
			Your friends from South Asia would support you to receive COVID-19 vaccination		148 (60.4)		
			Subjective Norm Scale^c^, mean (SD)		7.4 (1.6)		
		**Perceived behavioral control related to COVID-19 vaccination (agree)**					
			Receiving COVID-19 vaccination is easy for you if you want to		188 (76.7)		
			You are confident to receive COVID-19 vaccination in the next 6 months if you want to		149 (60.8)		
			Perceived Behavioral Control Scale^d^, mean (SD)		5.2 (1.1)		
	**Among South Asians you know who are living in Hong Kong, how many of them have already taken up COVID-19 vaccines?**						
		0/not sure			54 (22.0)		
		1-2			34 (13.9)		
		3-5			51 (20.8)		
		6-10			29 (11.8)		
		>10			77 (31.4)		
**Interpersonal-level factors, n (%)**							
	**Frequency of exposure to the following information related to COVID-19 vaccination on social media (sometimes/always)**						
		Positive information related to COVID-19 vaccination (eg, promising efficacy of the vaccines, new vaccines will enter the market soon)			151 (61.6)		
		COVID-19 vaccination will cause deaths and other serious conditions			120 (49.0)		
		Many people in Hong Kong did not turn up for their appointment to receive COVID-19 vaccination			109 (44.5)		
	**Influence of peers**						
		**Did you hear about any South Asians who experienced serious side effects after taking up COVID-19 vaccines?**					
			No		188 (76.7)		
			Yes		57 (23.3)		
**Sociostructural-level factors** **, n (%)**							
	**Cultural and religious reasons for COVID-19 vaccination hesitancy (agree)**						
		You are concerned about the halal status of the COVID-19 vaccines				66 (26.9)	
		You are concerned that COVID-19 vaccines may not work well among South Asians, as they are developed by China and western countries				54 (22.0)	
		The body is sacred; it should not receive certain chemicals or blood or tissues from animals				61 (24.9)	
		COVID-19 should be healed by God or natural means				54 (22.0)	
		Taking up vaccination is violating God’s will				12 (4.9)	
		Cultural and Religious Barrier Scale^e^, mean (SD)				8.3 (2.5)	
Level of confidence in Hong Kong’s health system, mean (SD)							7.2 (2.1)
Level of trust of the Hong Kong government regarding COVID-19 control, mean (SD)							7.7 (2.1)

^a^Positive Attitude Scale, 5 items, Cronbach α=.77, one factor was identified by exploratory factor analysis, explaining for 52.3% of the total variance.

^b^Negative Attitude Scale, 6 items, Cronbach α=.61, one factor was identified by exploratory factor analysis, explaining for 47.8% of the total variance.

^c^Subjective Norm Scale, 3 items, Cronbach α=.60, one factor was identified by exploratory factor analysis, explaining for 56.9% of the total variance.

^d^Perceived Behavioral Control Scale, 2 items, Cronbach α=.63, one factor was identified by exploratory factor analysis, explaining for 73.0% of the total variance.

^e^Cultural and Religious Barrier Scale, 5 items, Cronbach α=.65, one factor was identified by exploratory factor analysis, explaining for 42.2% of the total variance.

### Individual-, Interpersonal-, and Sociostructural-Level Variables Related to COVID-19 Vaccination

The Cronbach α of the scales based on the TPB ranged from .60 to .77; single factors were identified by exploratory factor analysis, explaining for 47.8%-73% of the total variance. Among the participants, 31.4% (77/245) had at least 10 peers who had received COVID-19 vaccination. About half of the participants were sometimes/always exposed to the following COVID-19 vaccination-related information on social media such as positive information (eg, promising efficacy of the vaccines, and new vaccines will enter the market soon) (151/245, 61.6%), COVID-19 vaccination will cause deaths and other serious conditions (120/245, 49.0%), and many people in Hong Kong did not turn up for their appointment to receive COVID-19 vaccination (109/245, 44.5%). Regarding the sociostructural-level variables, the Cronbach α of the Cultural and Religious Barrier Scale was .65; one factor was identified by exploratory factor analysis, explaining for 42.2% of the total variance (Table 2).

### Factors Associated With COVID-19 Vaccine Uptake

In univariate analysis, age group, ethnicity, relationship status, status as Hong Kong permanent residents, facemask wearing in public spaces/transportations other than workplaces, sanitizing hands after returning from public spaces or touching public installation, and avoiding social/meal gatherings with other people who do not live together were associated with COVID-19 vaccine uptake (Table 3).

**Table 3:** Associations between background characteristics and COVID-19 vaccine uptake among the South Asians who completed the web-based survey in Hong Kong on May 1-31, 2021 (N=245).

				Participants who had taken the COVID-19 vaccine (n=81), n (%)		Participants who had not taken the COVID-19 vaccine (n=164), n (%)		Crude odds ratio (95% CI)		*P* value	
**Sociodemographic data**											
	**Age group (years)**										
		18-29	10 (12.3)		73 (44.5)		1.0		N/A^a^		
		30-39	42 (51.9)		58 (35.4)		5.29 (2.45-11.43)		<.001		
		40-49	27 (33.3)		28 (17.1)		7.04 (3.02-16.41)		<.001		
		≥50	2 (2.5)		5 (3.0)		2.92 (0.50-17.11)		.24		
	**Gender**										
		Male	24 (29.6)		59 (36.0)		1.0		N/A		
		Female	57 (70.4)		105 (64.0)		1.34 (0.75-2.37)		.32		
	**Relationship status**										
		Currently single	20 (24.7)		70 (42.7)		1.0		N/A		
		Married or cohabited with a partner	61 (75.3)		94 (57.3)		2.27 (1.26-4.11)		.007		
	**Ethnicity**										
		Indian	33 (40.7)		50 (30.5)		1.0		N/A		
		Pakistani	16 (19.8)		73 (44.5)		0.33 (0.17-0.67)		.002		
		Nepali	25 (30.9)		27 (16.5)		1.40 (0.70-2.82)		.34		
		Other ethnicity groups	7 (8.6)		14 (8.5)		0.76 (0.28-2.08)		.59		
	**Permanent residents of Hong Kong**										
		Yes	36 (44.4)		36 (22.0)		1.0		N/A		
		No	45 (55.6)		128 (78.0)		0.35 (0.20-0.62)		<.001		
	**Highest education level attained**										
		Junior high or below	11 (13.6)		24 (16.4)		1.0		N/A		
		Senior high or equivalent	15 (18.5)		49 (29.9)		0.67 (0.27-1.67)		.39		
		College or university	38 (46.9)		65 (39.6)		1.28 (0.56-2.89)		.56		
		Postgraduate	17 (21.0)		26 (15.9)		1.43 (0.56-3.65)		.46		
	**Employment status**										
		Full-time	32 (39.5)		53 (32.3)		1.0		N/A		
		Others	49 (60.5)		111 (67.7)		0.73 (0.42-1.27)		.27		
	**Family members living with the participant**										
		0	7 (8.6)		8 (4.9)		1.0		N/A		
		1-2	19 (23.5)		25 (15.2)		0.87 (0.27-2.82)		.81		
		3-4	39 (48.1)		85 (51.8)		0.52 (0.18-1.55)		.24		
		≥5	16 (19.8)		46 (28.0)		0.40 (0.12-1.27)		.12		
	**Living with an older adult aged ≥60 years**										
		No	61 (75.3)		120 (73.2)		1.0		N/A		
		Yes	20 (24.7)		44 (26.8)		0.89 (0.49-1.65)		.72		
	**Living with a child younger than 18 years**										
		No	17 (21.0)		52 (31.7)		1.0		N/A		
		Yes	64 (79.0)		112 (68.3)		1.75 (0.93-3.28)		.08		
	**Having at least one chronic condition**										
		No	74 (91.4)		147 (89.6)		1.0		N/A		
		Yes	7 (8.6)		17 (10.4)		0.82 (0.33-2.06)		.62		
**Compliance to personal preventive behaviors and physical distancing**											
	**Frequency of facemask wearing when in proximity with other people in workplace**										N/A
		Never/sometimes/often	10 (12.3)		36 (22.0)		1.0				
		Every time	71 (87.7)		128 (78.0)		2.00 (0.94-4.26)		.07		
	**Frequency of facemask wearing in public spaces/transportations other than workplaces**										
		Never/sometimes/often	1 (1.2)		19 (11.6)		1.0		N/A		
		Every time	80 (98.8)		145 (88.4)		10.48 (1.38-79.76)		.02		
	**Sanitizing hands after returning from public spaces or touching public installation**										
		Never/sometimes/often	13 (16.0)		47 (28.7)		1.0		N/A		
		Every time	68 (84.0)		117 (71.3)		2.10 (1.06-4.16)		.03		
	**Avoiding social/meal gathering with other people who do not live together**										
		No	15 (18.5)		50 (30.5)		1.0		N/A		
		Yes	66 (81.5)		114 (69.5)		1.93 (1.01-3.70)		.048		
	**Avoiding crowded places**										
		No	14 (17.3)		45 (27.4)		1.0		N/A		
		Yes	67 (82.7)		119 (72.6)		1.81 (0.93-3.54)		.08		

^a^N/A: not applicable.

After adjusting for significant background characteristics, perceived higher cultural and religious barriers to receive COVID-19 vaccination were associated with lower COVID-19 vaccine uptake (AOR 0.83, 95% CI 0.71-0.97; *P*=.02). At the individual level, having more positive attitudes toward COVID-19 vaccination (AOR 1.31, 95% CI 1.10-1.55; *P*=.002), perceived support from significant others (AOR 1.29, 95% CI 1.03-1.60; *P*=.03), and perceived higher behavioral control to receive COVID-19 vaccination (AOR 2.63, 95% CI 1.65-4.19; *P*<.001) were associated with higher COVID-19 vaccine uptake, while a negative association was found between negative attitudes and the dependent variable (AOR 0.73, 95% CI 0.62-0.85; *P*<.001). Knowing more peers who had received COVID-19 vaccination was also associated with higher uptake (AOR 1.39, 95% CI 1.11-1.74; *P*=.01). At the interpersonal level, higher exposure to information about deaths and other serious conditions caused by COVID-19 vaccination was associated with lower uptake (AOR 0.54, 95% CI 0.33-0.86; *P*=.01) (Table 4).

**Table 4:** Factors associated with COVID-19 vaccine uptake among the South Asians who completed the web-based survey in Hong Kong on May 1-31, 2021.

Factors					Participants who had taken the COVID-19 vaccine (n=81)		Participants who had not taken the COVID-19 vaccine (n=164)		Crude odds ratio (95% CI)		*P* value		Adjusted odds ratio^a^ (95% CI)		*P* value
**Individual-level factors, mean (SD)**															
	Positive Attitude Scale			13.6 (1.7)		12.2 (2.5)		1.31 (1.17-1.56)		<.001		1.31 (1.10-1.55)		.002	
	Negative Attitude Scale			10.9 (2.1)		12.4 (2.3)		0.74 (0.64-0.84)		<.001		0.73 (0.62-0.85)		<.001	
	Subjective Norm Scale			7.9 (1.3)		7.1 (1.7)		1.47 (1.21-1.69)		<.001		1.29 (1.03-1.60)		.03	
	Perceived Behavioral Control Scale			5.7 (0.7)		4.9 (1.2)		2.70 (1.82-4.00)		<.001		2.63 (1.65-4.19)		<.001	
	Among South Asians you know who are living in Hong Kong-how many of them have already taken up COVID-19 vaccines?			3.9 (1.5)		2.8 (1.5)		1.60 (1.32-1.95)		<.001		1.39 (1.11-1.74)		.01	
**Interpersonal-level factors**															
	**Frequency of exposure to the following information related to COVID-19 vaccination on social media, mean (SD)**														
		Positive information related to COVID-19 vaccination		1.8 (0.8)		1.6 (0.9)		1.32 (0.95-1.82)		.10		1.16 (0.78-1.72)		.46	
		COVID-19 vaccination will cause deaths and other serious conditions		1.3 (0.6)		1.5 (0.8)		0.67 (0.46-0.98)		.04		0.54 (0.33-0.86)		.01	
		Many people in Hong Kong did not turn up for their appointment to receive COVID-19 vaccination		1.3 (0.8)		1.3 (0.8)		0.99 (0.71-1.37)		.94		0.98 (0.66-1.56)		.92	
	**Influence of peers, n (%)**														
		**Did you hear about any South Asians who experienced serious side effects after taking up COVID-19 vaccines?**													
			No	65 (80.2)		123 (75.0)		1.0		N/A^b^		1.0		N/A	
			Yes	16 (19.8)		41 (25.0)		0.74 (0.39-1.42)		.36		0.69 (0.31-1.52)		.35	
**Sociostructural-level factors, mean (SD)**															
	Cultural and Religious Barrier Scale			7.5 (2.1)		8.7 (2.6)		0.81 (0.72-0.91)		.001		0.83 (0.71-0.97)		.02	
	Level of confidence in Hong Kong’s health system			7.4 (2.4)		7.2 (2.0)		1.06 (0.93-1.21)		.36		0.95 (0.82-1.11)		.53	
	Level of trust of the Hong Kong government regarding COVID-19 control			8.2 (1.9)		7.5 (2.2)		1.18 (1.03-1.36)		.02		1.09 (0.92-1.29)		.33	

^a^Odds ratio adjusted for significant background characteristics (age group, relationship status, ethnicity, permanent residents of Hong Kong, frequency of facemask wearing in public spaces/transportation other than workplaces, sanitizing hands after returning form public spaces or touching public installation, and avoiding social/meal gathering with other people who do not live together).

^b^N/A: not applicable.

## Discussion

To our knowledge, this is one of the first studies investigating the determinants of COVID-19 vaccine uptake by using the socioecological perspective among ethnic minorities. About one-third of the participants received at least one dose of COVID-19 vaccination. Factors at the individual level (perceptions related to COVID-19 vaccination), interpersonal level (influence of social media), and sociostructural level (cultural belief) were determinants of the COVID-19 vaccine uptake. Using the socioecological model allows us to understand COVID-19 vaccination behaviors from a comprehensive perspective.

Participants aged 30-49 years reported the highest COVID-19 vaccine uptake; this finding was different from that reported in the Chinese population [23-26]. Married or cohabitation with a partner was also associated with higher COVID-19 vaccine uptake. Future studies should confirm whether protecting one’s partner is the motivation to receive COVID-19 vaccination. In addition, South Asians who were not Hong Kong permanent residents reported lower COVID-19 vaccine uptake. Nonpermanent residents might be less familiar with the health care system in Hong Kong and should receive more support in future programs. As for COVID-19 preventive behaviors, similar to that reported in previous studies [19,22], higher compliance to personal preventive behaviors (eg, consistent facemask wearing in public spaces and hand hygiene) and physical distancing behaviors (eg, avoiding social/meal gathering) was associated with higher uptake.

Culturally, our findings also showed that Pakistanis reported significantly lower COVID-19 vaccine uptake compared to Indians. Differences in the religious belief might partially explain this variation. Islam is the major religion for Pakistanis, and news reported concerns about the halal status of COVID-19 vaccines among Muslims [13]. Our findings suggested that some unique strategies should be tailored for South Asians to address cultural and religious reasons for COVID-19 vaccination hesitancy. The results confirmed that concerns about the halal status of COVID-19 vaccines was a barrier for South Asians to take up the vaccines [15]. The government should work with the vaccine manufacturers to clarify whether gelatin, which has been commonly used as a stabilizer for the safety and effectiveness of vaccines during storage and transportation, is a part of the available COVID-19 vaccines. In order to address other cultural or religious issues, future programs should engage community groups, champions and faith leaders, and develop culturally competent interventions.

At the individual level, South Asians shared some similar facilitators as the local Chinese population to receive COVID-19 vaccination, including perceived efficacy of COVID-19 vaccination in protecting themselves and their family members, perceived impacts of COVID-19 vaccination on pandemic control, perceived support from significant others, and perceived behavioral control of taking up COVID-19 vaccination [23-26]. Concerns about side effects and difficulties in choosing the most suitable COVID-19 vaccine were common barriers [23-26]. Therefore, the same health promotion strategies might be useful for both South Asians and Chinese in Hong Kong. In order to prevent the choice overload [21], efficacies and side effects of different COVID-19 vaccines available in Hong Kong can be compared on a table, which makes it easy for participants to compare features across products [21]. Laymen’s terms in participants’ native language should be used to emphasize that severe side effects of COVID-19 vaccination are rare, and the pros of vaccination outweigh its cons. Testimonials of people who received different types of COVID-19 vaccines regarding side effects should be presented. Health communication messages should also demonstrate that the procedures to receive vaccination are easy and convenient to reduce concerns regarding vaccination procedures. The recommendation made by health care providers is a strong facilitator of COVID-19 vaccine uptake [23-26]. Performing outreach vaccination services in South Asian communities is also useful for enhancing their perceived behavioral control. The results also supported a significant influence of peers on COVID-19 vaccine uptake among South Asians. Updated information about how many South Asians had received COVID-19 vaccination should be disseminated to this group. At the interpersonal level, exposure to negative information about COVID-19 negatively affects vaccine uptake. Health authorities in Hong Kong should identify and address common misinformation in a timely manner. It is encouraging that the government has started to clarify this misinformation on the vaccination program webpage.

The findings of this study should be interpreted in light of some limitations. First, a direct measure of perceived behavioral control should assess self-efficacy and perceived controllability [29,30]. Previous studies have suggested that these 2 constructs were differently associated with behavioral intention and actual behaviors [29,30]. In this study, the scale measuring perceived behavioral control was adapted from a validated tool [19]. Owing to the limited length of the questionnaire, the measurement only had 2 items and mainly covered self-efficacy. Failure to measure perceived controllability together with self-efficacy was one major limitation of this study. Second, in the absence of a sampling frame, participants were conveniently recruited online. Similar to other web-based surveys, the response rate was relatively low (about 25%). We were not able to obtain the characteristics of the active WhatsApp contacts who did not respond to our invitation or refused to complete the survey. Therefore, we were not able to compare the characteristics of the respondents and nonrespondents. There was a selection bias. Generalizations should be made cautiously to South Asians in Hong Kong. Yet, because ethnic minorities is a special population in Hong Kong, using the traditional randomly sampling method of telephone or mail survey was not feasible for administering the questionnaire to this targeted population. Third, some items and scales used in this study were single items, self-constructed, or modified from those used in published studies in the general population. We purposely decided not to use standardized western scale measurements to account for the cultural variations experienced by the ethnic minorities in Hong Kong. A self-constructed or modified scale that is designed to suit the local context of Hong Kong is more suitable. Furthermore, the use of a single-item scale will significantly decrease the burden of the respondents who need to take time to complete the survey. Moreover, this was a cross-sectional study and could not establish causal relationships.

Using the socioecological model, this study offered an overview for us to identify tapping points that can encourage COVID-19 vaccine uptake among South Asian populations in Hong Kong. Because they shared cultural and social orientations dissimilar from those of the local Chinese in Hong Kong, current empirical evidence offered a guide on how promotional strategies can be customized for this population. We recommend religion-targeted, outreach community, and peer-based programs to enhance COVID-19 vaccine uptake rate among South Asians in Hong Kong. To further customize the promotion, these programs can be co-designed, shared, and endorsed by South Asian communities such as nonprofit groups, champions, and faith leaders to develop culturally competent interventions.

## Appendix

Multimedia Appendix 1 English version of the questionnaire survey.

## Abbreviations

AOR: adjusted odds ratio
CBO: community-based organization
TPB: Theory of Planned Behavior
