# Supplementary material for: Multilevel Determinants of COVID-19 Vaccine Uptake Among South Asian Ethnic Minorities in Hong Kong: Cross-sectional Web-Based Survey
Source: JMIR Public Health Surveill. 2021 Nov 9;7(11):e31707. doi: 10.2196/31707 (PMC8582756; doi:10.2196/31707)
Supplement: Multimedia Appendix 1 [file publichealth_v7i11e31707_app1.docx]

Study ID: __________

**Facilitators and barriers to receive COVID-19 vaccination among South Asians in Hong Kong**

Dear participants, the JC School of Public Health and Primary Care of the Chinese University of Hong Kong is conducting an online survey to understand facilitators and barriers to receive COVID-19 vaccination among South Asian ethnic minorities in Hong Kong. We sincerely invite you to participate. This research is anonymous, your information will only be used for scientific research purposes and will be kept strictly confidential. Participation is completely voluntary, and your refusal will have no consequences. Upon completion of this survey, a HK$50 supermarket coupon will be sent to you in a plain envelope without any information about this study.

**Part 1**

1-1 Have you ever taken up COVID-19 vaccines?

□_1_ Yes --- please answer 1-1A to 1-1D □_2_ No --- please answer 1-2

1-1A How many doses of COVID-19 vaccines did you take up

□_1_ 1 dose □_2_ 2 doses

1-1B Which type of COVID-19 vaccines did you take up

□_1_ Sinovac-Biotech □_2_ BioNTech-Fosun Pharama □_3_ Not sure

1-1C Where did you take up these COVID-19 vaccines?

□_1_ Community vaccination centers □_2_ Public hospitals (e.g., General out-patient clinics)

□_3_ Private clinics □_4_ Other settings, please be specific_______________

1-1D How severe are the side-effects of the COVID-19 vaccines?

□_1_ Not at all □_2_ Very mild □_3_ Mild □_4_ Moderate □_5_ Severe □_6_ Very severe

1-2 Do you agree with the following statements related to COVID-19 vaccination, there is no right or wrong answers

|  | **Disagree** | **Neutral** | **Agree** |
| --- | --- | --- | --- |
| A COVID-19 vaccination is highly effective in protecting you from COVID-19 | 1 | 2 | 3 |
| B Taking up COVID-19 vaccination is highly effective in protecting your family members against COVID-19 | 1 | 2 | 3 |
| C Taking up COVID-19 vaccination can facilitate resumption of cross-boundary travelling | 1 | 2 | 3 |
| D Taking up COVID-19 vaccination can contribute to the control of COVID-19 in Hong Kong | 1 | 2 | 3 |
| E Hong Kong will have adequate supply of COVID-19 vaccination | 1 | 2 | 3 |
| F COVID-19 vaccines will have severe side-effects | 1 | 2 | 3 |
| G The side-effects of COVID-19 vaccines in long run is unclear | 1 | 2 | 3 |
| H The protection of COVID-19 vaccines will only last for a short time | 1 | 2 | 3 |
| I It is difficult for you to register for COVID-19 vaccination | 1 | 2 | 3 |
| J These is a lack of information related to the COVID-19 vaccination program in my mother tongue | 1 | 2 | 3 |
| K You do not know which type of COVID-19 vaccine is most suitable for you | 1 | 2 | 3 |
| L Doctors and nurse would support you to receive COVID-19 vaccination | 1 | 2 | 3 |
| M Your family members will support you to receive COVID-19 vaccination | 1 | 2 | 3 |
| N Your friends from South Asia would support you to receive COVID-19 vaccination | 1 | 2 | 3 |
| O Receiving COVID-19 vaccination is easy for you if you want to | 1 | 2 | 3 |
| P You are confident to receive COVID-19 vaccination in the next six months if you want to | 1 | 2 | 3 |

1-3 Do you agree with the following statements (cultural belief/barriers related to vaccination)

|  | **Disagree** | **Neutral** | **Agree** |
| --- | --- | --- | --- |
| A. You are concerned about the halal status of the COVID-19 vaccines | 1 | 2 | 3 |
| B. You are concerned about that COVID-19 vaccines may not work well among South Asians, as they are developed by China and western countries | 1 | 2 | 3 |
| C. The body is sacred, should not receive certain chemicals or blood or tissues from animals | 1 | 2 | 3 |
| D. COVID-19 should be healed by God or natural means | 1 | 2 | 3 |
| E. Taking up vaccination is violating God’s will | 1 | 2 | 3 |

1-4 Frequency of exposure to the following information related to COVID-19 vaccination on social media (Facebook, Twitter, Flicker, Tiktok, etc.) in the past month

|  | **Almost never** | **Seldom** | **Sometimes** | **Always** |
| --- | --- | --- | --- | --- |
| A. Positive information related to COVID-19 vaccination (e.g., new vaccines entering clinical trials, promising efficacy of the vaccines, and vaccines, and vaccines will enter the market soon) | 0 | 1 | 2 | 3 |
| B. COVID-19 vaccination will cause deaths and other serious conditions | 0 | 1 | 2 | 3 |
| C. Many people in Hong Kong did not turn up for their appointment to receive COVID-19 vaccination | 0 | 1 | 2 | 3 |

1-5 Among South Asians you know who are living in Hong Kong, how many of them have already taken up COVID-19 vaccines?

□_1_ None □_2_ 1-2 □_3_ 3-5 □_4_ 6-10 □_5_ More than 10 □_6_ Not sure

1-6 Did you hear about any South Asians who experienced serious side-effects after taking up COVID-19 vaccines?

□_1_ No □_2_ Yes

**Part 2 Personal preventive measures**

2-1 Frequency of facemask wearing in public places/transportations other than workplaces in the past month

□_1_ Every time □_2_ Often □_3_ Sometimes □_4_ Never

2-2 Frequency of facemask wearing when you have close contact with other people in workplace in the past month

□_1_ Every time □_2_ Often □_3_ Sometimes □_4_ Never □_5_ No applicable

2-3 Frequency of sanitizing hands (using soaps, liquid soaps or alcohol-based sanitizer) after returning from public spaces or touching public installation

□_1_ Every time □_2_ Often □_3_ Sometimes □_4_ Never

| 2-4 In the past month, did you…… | **Yes** | **No** |
| --- | --- | --- |
| A. Avoid social/meal gathering with other people who do not live together | 1 | 2 |
| B. Avoid crowed places | 1 | 2 |

**Part 3 Structural barriers and trust in health system**

3-1 How much confidence do you have in Hong Kong’s healthcare system? Please rate it between 1 (not at all) to 10 (extremely confident). Your answer is [ ]

3-2 How much do you trust the Hong Kong government regarding COVID-19 control? Please rate it between 1 (not at all) to 10 (extremely confident). Your answer is [ ]

**Part 4** **Socio demographic information**

4-1 What is your age?

□_1_ 18-25 □_2_ 25-29 □_3_ 30-39 □_4_ 40-49 □_5_ 50-59 □_6_ 60-69

□_7_ 70-79 □_8_ 80-85 □_9_ Above 85

4-2 What is your gender? □_1_ Male □_2_ Female □_3_ Others

4-3 What is your relationship status?

□_1_ Single □_2_ Married or cohabited with a man/woman □_3_ Divorced □_4_ Widowed

4-4 How many family members are living with you?

□_1_ 0 □_2_ 1-2 □_3_ 3-4 □_4_ 5-10 □_5_ >10

4-5 What is your ethnicity?

□_1_ Indian □_2_ Pakistani □_3_ Nepali □_4_ Sri Lankans □_5_ Other ethnicity group

□_6_ Jainism □_7_ Others □_3_ Refuse to answer

4-6 Are you a Hong Kong permanent resident?

□_1_ Yes □_2_ No --- please answer 4-7A

4-7 Your education level is:

□_1_ Junior high or below □_2_ Senior high or equivalent □_3_ College or university □_4_ Postgraduate

4-8 What is your current employment status?

□_1_ Full-time □_2_ Part-time □_3_ Self-employed □_4_ Housewife □_5_ Unemployed

□_6_ Retired □_7_ Students □_8_ Others

4-9 Are you diagnosed with chronic disease by doctors (such as hypertension, diabetes, chronic lung disease, heart infarction, liver disease?) □_1_ Yes □_2_ No

4-10 Are there any children under 18 in your family □_1_ Yes □_2_ No

4-11 Are there any elderly people 60 years of age or older in the family □_1_ Yes □_2_ No

**End of the questionnaire**
